# Development and validation of a predictive model for depression risk in patients with amyotrophic lateral sclerosis

**DOI:** 10.3389/fneur.2025.1639895

**Published:** 2025-09-11

**Authors:** Man Liu, Tongyang Niu, Xinyi Zhang, Ziyao Zhang, Luqi Zhao, Jiaqi Li, Siyu Fu, Meiqi Han, Rui Li, Hui Dong, Yaling Liu

**Affiliations:** ^1^Department of Neurology, The Second Hospital of Hebei Medical University, Shijiazhuang, Hebei, China; ^2^Key Laboratory of Clinical Neurology, Ministry of Education, Hebei Medical University, Shijiazhuang, Hebei, China; ^3^Neurological Laboratory of Hebei Province, Shijiazhuang, Hebei, China

**Keywords:** amyotrophic lateral sclerosis, clinical impact curve, depression, model validation, nomogram

## Abstract

**Introduction:**

Depression is a severe neuropsychiatric manifestation in patients with amyotrophic lateral sclerosis (ALS), substantially impacting their quality of life and exacerbating caregiver burden, due to the need for different approaches in clinical care. However, a predictive model for the risk of depression in patients with ALS is lacking. This study aimed to develop and validate a predictive model using routinely accessible clinical and laboratory indicators to identify patients at high risk of depression.

**Methods:**

Patients with ALS who were hospitalized in the Department of Neurology at the Second Hospital of Hebei Medical University between March 2017 and December 2024 were included. Basic clinical data, laboratory test results, and relevant questionnaire scores were collected, and patients were divided into depressed and non-depressed groups. The least absolute shrinkage and selection operator regression and multivariate logistic regression analyses were applied for variable selection and model construction. Model performance was evaluated using the area under the receiver operating characteristic curve, calibration curves, decision curve analysis, and clinical impact curves, with internal validation performed via bootstrap resampling.

**Results:**

Depression was observed in 33.9% of patients. Significant predictors included educational level, sleep disorders, anxiety, Revised Amyotrophic Lateral Sclerosis Functional Rating Scale total scores, C-reactive protein levels, and the Systemic Inflammation Response Index. The final model demonstrated good predictive accuracy and clinical applicability. A depression risk scoring table was further developed based on the coefficients of the logistic regression.

**Conclusion:**

The nomogram and the scoring table offer a reliable and practical approach for clinicians to identify patients with ALS who are at high risk for depression and enable early psychological intervention in clinical settings.

## 1 Introduction

Amyotrophic lateral sclerosis (ALS) is a rare neurodegenerative disease that primarily affects the motor system. It is characterized by progressive degeneration of upper and lower motor neurons, resulting in muscle paralysis and ultimately death, with no available cure (1). ALS is a rapidly progressive disease. Although its incidence is relatively low, at approximately 2 cases per 100,000 people, its mean survival period ranges from 2 to 3 years from diagnosis (2).

In addition to motor decline, approximately 50% of patients experience extra-motor or non-motor symptoms, including disturbances in emotional processing, cognition, behavior, depression, and anxiety (3). Depression is one of the most severe neuropsychiatric manifestations, with clinical features such as low mood, low energy, poor concentration, inappropriate guilt, changes in appetite, insomnia or hypersomnia, irritability, and thoughts of death (4). ALS is associated with a significantly increased risk of depression (5–7). Potential contributing mechanisms include the psychological burden of an incurable disease (6); cortical thinning in the prefrontal cortex and other brain regions; disruption of mood-related brain networks; dysfunction of neurotransmitter systems; altered cortisol levels (3, 8); and, potentially, neuroinflammation as a shared pathophysiological mechanism in both ALS and depression (9–11). Notably, ALS may obscure or mask depressive symptoms due to clinical overlap, leading to underdiagnosis (12). The variability and subtlety of depressive presentations, combined with the subjective nature of assessments, often complicate clinical identification (13).

Despite the availability of medical interventions for comorbid depression in ALS, a delay in its diagnosis can be severely detrimental. Depression can seriously impair the quality of life of patients with ALS, affecting their physical condition, vitality, social participation, emotional functioning, and mental well-being (14). Moreover, depression worsens clinical outcomes, accelerates disease progression, and is a major contributor to disability (15). Additionally, disparities in clinical care can further increase the burden on caregivers (16). Therefore, it is essential to proactively identify patients with ALS who are at risk of depression and implement preventive strategies.

To date, no predictive model has been established to assess depression risk in patients with ALS. We aimed to develop a clinically applicable nomogram and scoring table that incorporate routinely accessible clinical and laboratory indicators to predict the likelihood of depression in patients with ALS. The model may facilitate early identification of high-risk individuals and enable timely interventions, potentially improving both quality of life and survival outcomes.

## 2 Materials and methods

### 2.1 Patient selection

This retrospective case–control study included patients with ALS who were hospitalized in the Department of Neurology at the Second Hospital of Hebei Medical University between March 2017 and December 2024. Eligible participants met all of the following criteria: (i) a diagnosis of possible, probable, or definite ALS according to the El Escorial Criteria; (ii) age of 18–80 years; (iii) preserved pulmonary function (exertion spirometry >85%); and (iv) no cognitive impairment, as indicated by standardized scores on both the Montreal Cognitive Assessment and the Brief Mental State Examination.

**Figure 1:**
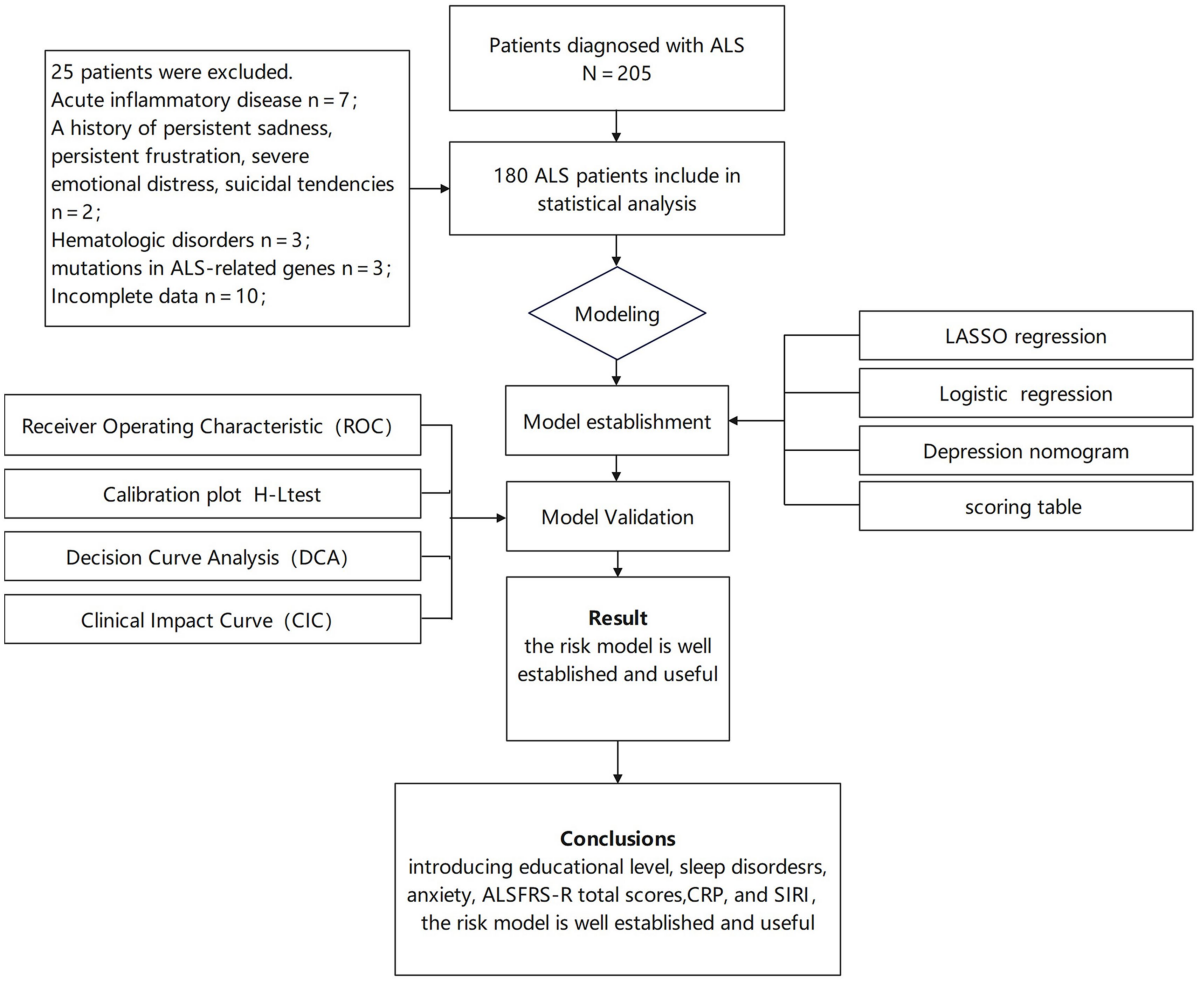
Flow diagram of study design.

Exclusion criteria were as follows: mutations in ALS-related genes (for example, *SOD1*, *C9ORF72*, *FUS*, and *TDP-43*); a diagnosis of frontotemporal lobe dementia; existing psychotic disorders; patients with a history of persistent sadness, persistent frustration, severe emotional distress, suicidal tendencies; comorbid metabolic disorders, neoplastic diseases, severe acute inflammatory conditions, or hematologic abnormalities; patients for whom data for the collected indicators were missing; and current pregnancy or pregnancy in the preceding 6 months. Finally, 180 patients were included in the study (Figure 1).

The study protocol was approved by the Institutional Review Board of the Second Hospital of Hebei Medical University. The study was conducted in accordance with the ethical principles outlined in the Declaration of Helsinki. All participants provided written informed consent after being fully informed of the study’s aims and procedures.

### 2.2 Research methods

Relevant literature and clinical guidelines on depression were reviewed, and previously reported factors associated with depression were identified (9, 17–19). Based on the authors’ clinical experience, these factors were compiled and analyzed. Depression was assessed using the Hospital Anxiety and Depression Scale (HADS), with a score >7 indicating the presence of depression (20). In a quiet and relaxed environment, patients independently completed the HADS under the guidance of a qualified neurologist. The doctor verified the completeness of the questionnaire, and any missing items were completed immediately. If a patient was unable to complete it independently (e.g., due to severe limb paralysis, inability to read or write, etc.) the scale was completed with assistance from a physician.

Data collected included demographic and clinical characteristics, such as age, sex, body mass index (BMI), smoking and alcohol use history, marital status, and educational level (completion of at least 9 years of compulsory education). ALS-specific clinical variables included site of onset, disease duration, Pittsburgh Sleep Quality Index (PSQI) score, C-reactive protein (CRP) level, neutrophil-to-lymphocyte ratio (NLR), monocyte-to-lymphocyte ratio (MLR), platelet-to-lymphocyte ratio (PLR), systemic immune-inflammation index (SII), and systemic inflammation response index (SIRI).

The SII was calculated as follows:

SII = (neutrophil count × platelet count)/lymphocyte count

The SIRI was calculated as follows:

SIRI = (neutrophil count × monocyte count)/lymphocyte count.

Amyotrophic lateral sclerosis disease severity was assessed using the Revised Amyotrophic Lateral Sclerosis Functional Rating Scale (ALSFRS-R). Anxiety was evaluated using the HADS, with scores >7 indicating anxiety. Sleep quality was measured using the PSQI, with scores >6 indicating sleep disorders.

### 2.3 Statistical analyses

All statistical analyses were performed using IBM SPSS Statistics (Version 26.0; IBM Corp., Armonk, NY, United States) and R software (version 4.2.0; R Core Team). Continuous variables with a normal distribution were reported as means ± standard deviations, while non-normally distributed variables were expressed as medians and interquartile ranges. Categorical variables were summarized as frequencies and percentages.

Comparisons between groups were performed using the independent samples *t*-test or rank-sum test for continuous variables, and the chi-square test for categorical variables. Variable selection was conducted using the least absolute shrinkage and selection operator (LASSO) regression to identify optimal predictors. Variables selected by LASSO were entered into a multivariate logistic regression model to identify independent predictors of depression (*p* < 0.05).

Subsequently, a predictive nomogram was constructed based on the significant predictors. The discriminatory performance of the nomogram was evaluated using the area under the receiver operating characteristic (ROC) curve (AUC). Calibration was assessed using calibration plots and the Hosmer–Lemeshow goodness-of-fit test to determine agreement between observed outcomes and model predictions. The clinical utility of the model was further assessed using decision curve analysis (DCA) and clinical impact curves. The results of univariate and logistic regression analyses were combined and a risk scoring table for depression in ALS was constructed based on the Framingham risk score model.

## 3 Results

### 3.1 Characteristics of patients with ALS with or without depression

A total of 180 patients included during the study period were divided into two groups based on their HADS scores: depression (*n* = 61) or non-depression (*n* = 119), yielding a depression prevalence of 33.9%. Patients with depression had significantly higher rates of anxiety, elevated CRP levels, and increased SIRI and MLR values than patients without depression. They were less likely to have completed 9 years of compulsory education. Additionally, depression was associated with a higher prevalence of sleep disorders and lower ALSFRS-R total scores (Table 1).

**Table 1:** Characteristics of participants with ALS with or without depression.

Parameter	All individuals (*n* = 180)	Non-depression (*n* = 119)	Depression (*n* = 61)	*p*-value
Age (years)	61 (53, 68)	60 (53, 67)	63 (55, 70)	0.192
Gender				0.974
Female	72 (40%)	47 (39%)	25 (41%)	
Male	108 (60%)	72 (61%)	36 (59%)	
Smoking				0.38
No	127 (71%)	87 (73%)	40 (66%)	
Yes	53 (29%)	32 (27%)	21 (34%)	
Alcohol				0.536
No	126 (70%)	81 (68%)	45 (74%)	
Yes	54 (30%)	38 (32%)	16 (26%)	
BMI	24.03 (21.39, 26.68)	23.92 (21.69, 26.6)	24.22 (20.76, 26.73)	0.856
9 year compulsory education				0.032
No	76 (42%)	43 (36%)	33 (54%)	
Yes	104 (58%)	76 (64%)	28 (46%)	
Sleep disorders				< 0.001
No	87 (48%)	75 (63%)	12 (20%)	
Yes	93 (52%)	44 (37%)	49 (80%)	
Anxiety				< 0.001
No	147 (82%)	110 (92%)	37 (61%)	
Yes	33 (18%)	9 (8%)	24 (39%)	
Site of onset				0.543
Limb	119 (66%)	81 (68%)	38 (62%)	
Bulbar	61 (34%)	38 (32%)	23 (38%)	
Duration of disease	10 (6, 17)	9 (6, 15)	12 (6, 23)	0.529
ALSFRS-R total scores	39 (34, 43)	41 (36, 44)	35 (29, 38)	<0.001
CRP	2.38 (1.6, 4.2)	1.99 (1.52, 3.54)	3.16 (1.93, 8.4)	<0.001
NLR	1.96 (1.46, 2.51)	1.91 (1.46, 2.37)	2 (1.5, 2.92)	0.316
PLR	129.62 (103.89, 159.6)	130 (103.65, 156.75)	127.42 (105.78,163.38)	0.665
MLR	0.22 (0.17, 0.28)	0.22 (0.17, 0.26)	0.24 (0.18, 0.33)	0.041
SII	418.27 (308.09, 575.01)	411.7 (297.62, 553.37)	444.52 (332.3, 638.36)	0.081
SIRI	0.71 (0.48, 0.98)	0.65 (0.49, 0.87)	0.83 (0.48, 1.33)	0.008

### 3.2 LASSO regression and multivariate logistic regression analysis

**Figure 2:**
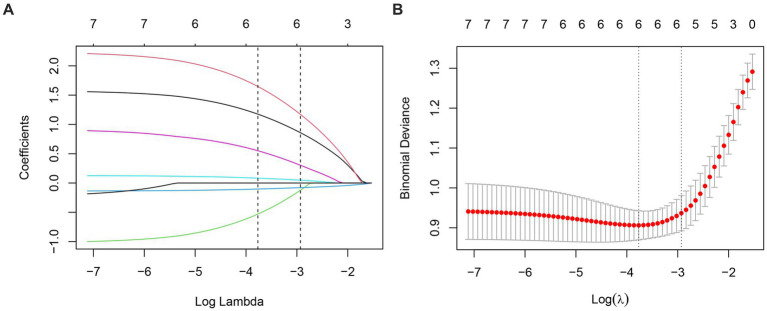
A coefficient profile was generated along the log (*λ*) sequence, with non-zero coefficients selected by the optimal λ **(A)**. Identification of optimal penalty coefficients in LASSO models through 10-fold cross-validation and minimum criterion **(B)**.

LASSO regression identified six variables with non-zero coefficients as significant predictors of depression: completion of 9 years of compulsory education, sleep disorders, anxiety, CRP levels, ALSFRS-R total scores, and SIRI values (Figures 2A,B). These predictors were subsequently included in a multivariate logistic regression analysis. The results of this analysis are presented in Table 2.

**Table 2:** Logistic regression analysis of predictors for depression.

Group	*B*	SE	Wald values	*p*-value	Odds ratio (95% CI)
9 year compulsory education					
NO					
YES	−1.033	0.449	5.306	0.021	0.356 (0.148–0.857)
Sleep disorders					
NO					
YES	1.586	0.470	11.370	0.001	4.886 (1.943–12.287)
Anxiety					
NO					
YES	2.243	0.551	16.568	< 0.001	9.419 (3.199–27.735)
ALSFRS-R total scores	−0.137	0.038	13.029	< 0.001	0.872 (0.809–0.939)
CRP	0.126	0.062	4.173	0.041	1.134 (1.005–1.280)
SIRI	0.877	0.401	4.776	0.029	2.403 (1.095–5.274)

### 3.3 Construction and validation of a nomogram for predicting depression in patients with ALS

**Figure 3:**
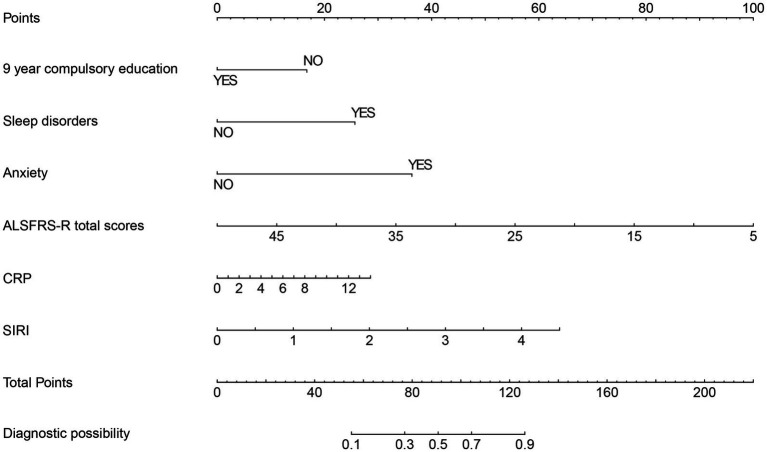
Nomogram for predicting depression in patients with ALS.

A nomogram was developed incorporating six predictive factors: completion of 9 years of compulsory education, sleep disorders, anxiety, ALSFRS-R total scores, CRP levels, and SIRI values. For each predictor, a perpendicular line was drawn from the observed value of a particular predictor to the “Points” axis to determine the corresponding score. The individual scores were summed to calculate a total point score, which was then used to project downward from the “Total Points” axis to the “Risk” scale, yielding the patient’s predicted probability of depression (Figure 3).

### 3.4 Discrimination performance of the prediction model

**Figure 4:**
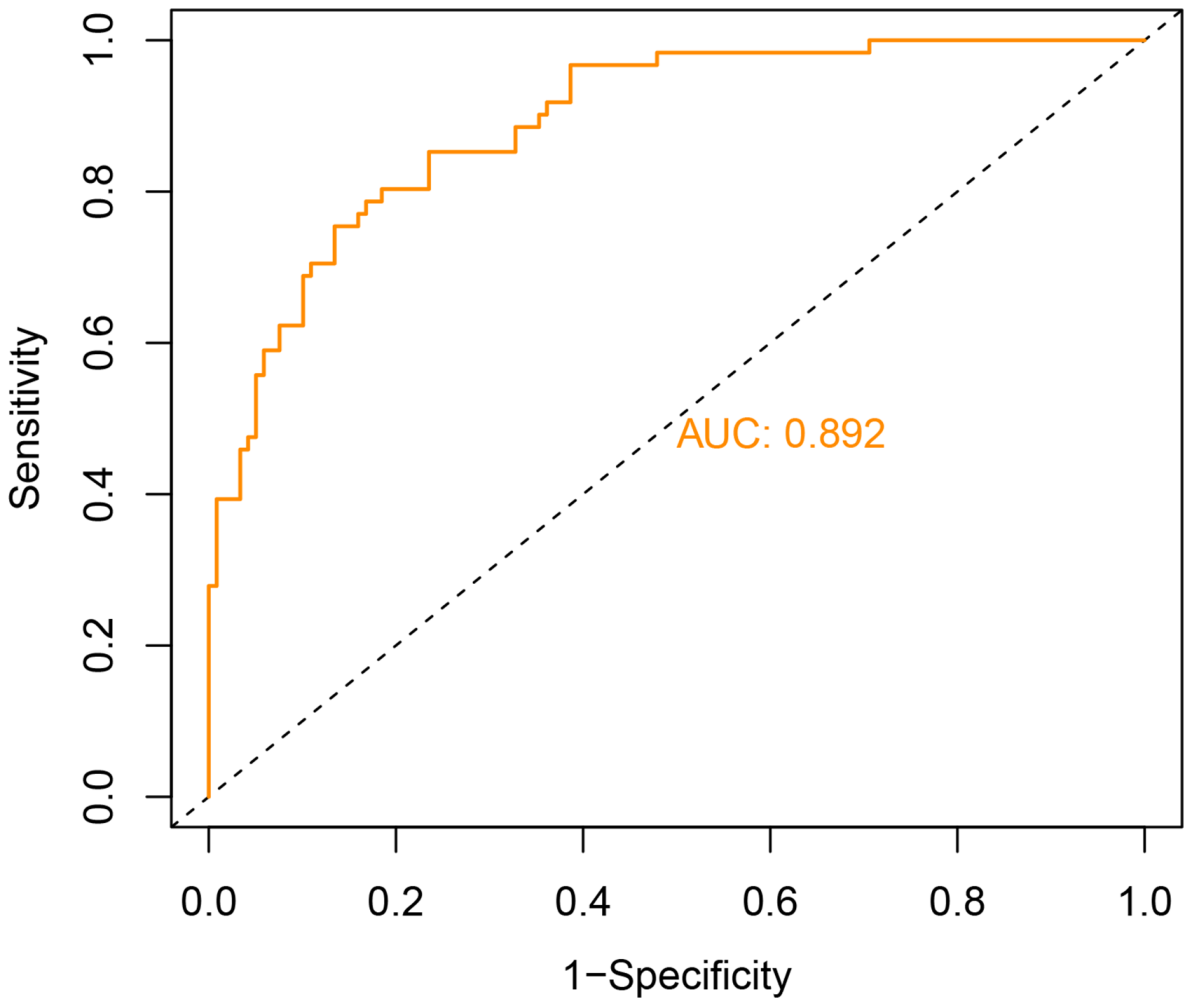
ROC curves for predicting depression in patients with ALS.

The model’s discriminative performance was assessed using ROC curve analysis. It demonstrated strong predictive ability, with an AUC of 0.892, indicating excellent discrimination (threshold >0.7). At an optimal cutoff value of 0.374, the model achieved a sensitivity of 75.4% and a specificity of 86.6% (Figure 4).

### 3.5 Calibration of the depression prediction model in patients with ALS

**Figure 5:**
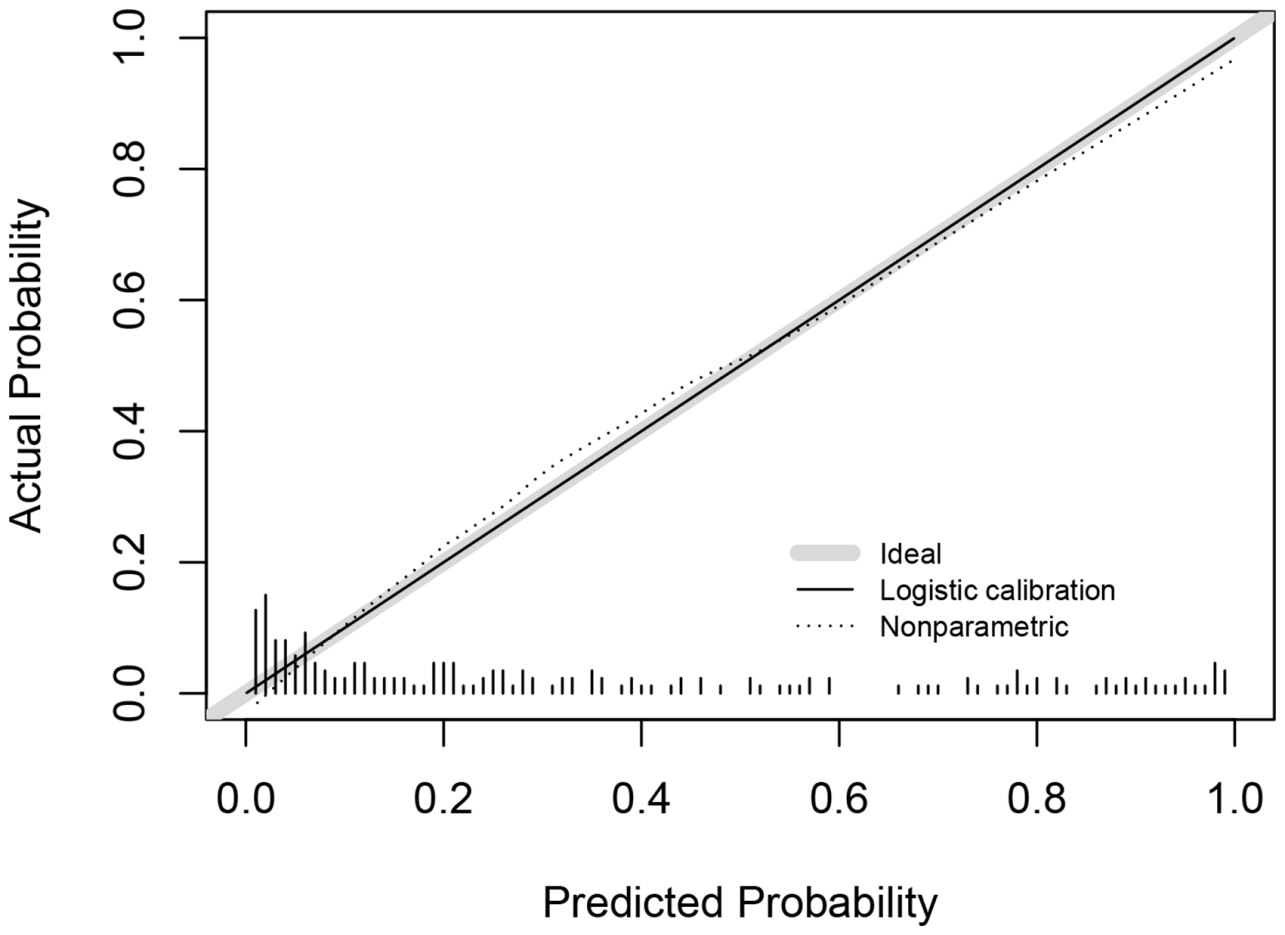
Calibration curves for predictive models.

Model calibration was assessed using a calibration curve and the Hosmer–Lemeshow test. Internal validation was conducted using 1,000 bootstrap resamples. The calibration curve closely followed the 45-degree reference line, indicating strong concordance between predicted and observed probabilities. The Hosmer–Lemeshow test yielded *p* = 0.284, supporting a good model fit (Figure 5).

### 3.6 Decision curve and clinical impact analyses

**Figure 6:**
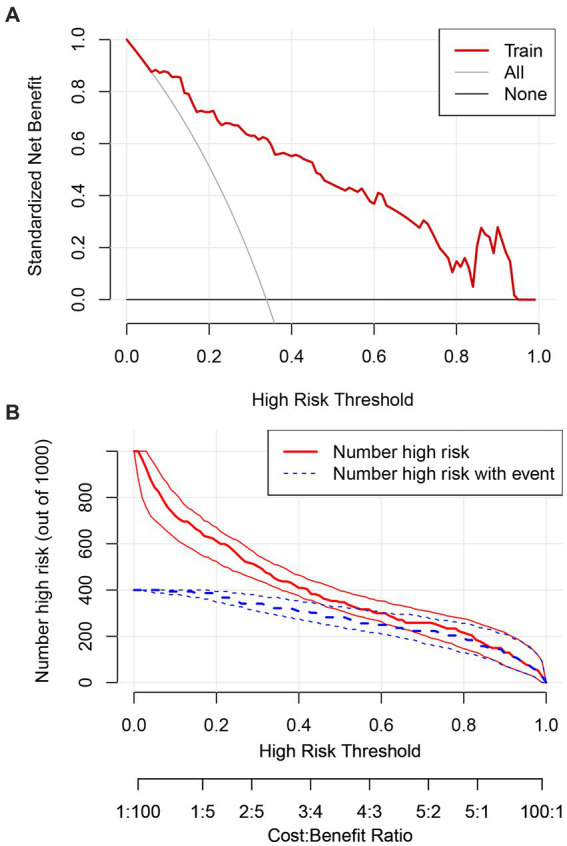
Decision curve analysis for the study model **(A)**. Clinical impact curve for the study model **(B)**.

The clinical utility of the prediction model was assessed using DCA and clinical impact curves. The DCA demonstrated that the model provided a net benefit across a wide range of threshold probabilities (8–95%), with the curve consistently exceeding the “Treat None” and “Treat All” strategies. The clinical impact curve further confirmed the model potential utility in clinical decision-making by demonstrating meaningful net benefit across relevant threshold ranges (Figures 6A,B).

### 3.7 Depression risk scoring table and predictive probability in patients with ALS

To facilitate rapid clinical assessment, we developed a depression risk scoring table based on the logistic regression coefficients presented in Table 2. For improved clinical applicability, the continuous variables, CRP levels and SIRI values, were categorized using optimal cutoff values: CRP (mg/L) as <2.89 or ≥2.89, and SIRI as <0.93 or ≥0.93. The resulting depression risk scores ranged from 0 to 17 (Table 3). The predicted probability values of risk corresponding to each score (Table 4) were calculated using the equations of the multivariate logistic regression model with the following formula:


$$\hat{P}=\frac{1}{1+e^{\left(-\sum_{i=0}^{p}\beta_{i}X_{i}\right)}},$$

$$\sum_{i=0}^{p}\beta_{i}X_{i}\approx \text{constant term}+\beta_{i}*W_{\mathrm{ij}}+B*\text{point total},$$

*w*_ij_ is the reference value for each group of influences and *B* is the constant corresponding to 1 in the scoring tool.


$$\sum_{i=0}^{p}\beta_{i}X_{i}=2.296+(-1.033)*1+1.586*0$$

$$\begin{array}{l} +2.243*0+(-0.137)*44+0.126*1.965+0.877\\ {}*0.545+0.685\;\text{point total} \end{array}$$

=−4.03945+0.685∗point total.

**Table 3:** Risk scoring table for depression in patients with ALS.

Influence factor	Category	Point
9 year compulsory education		
	No	2
	Yes	0
Sleep disorders		
	No	0
	Yes	2
Anxiety		
	No	0
	Yes	3
ALSFRS-R_total scores		
	0–19	7
	20–39	3
	40–48	0
CRP		
	<2.89	0
	≥2.89	1
SIRI		
	<0.93	0
	≥0.93	2

**Table 4:** Relationship between total score and predicted probability.

Point total	Estimate of risk
0	0.0173
1	0.0337
2	0.0648
3	0.1208
4	0.2143
5	0.3510
6	0.5176
7	0.6804
8	0.8085
9	0.8934
10	0.9432
11	0.9706
12	0.9849
13	0.9924
14	0.9961
15	0.9980
16	0.9990
17	0.9995

## 4 Discussion

Depression is a common non-motor symptom of ALS. Due to the variability of depressive symptom presentation, the subjective nature of assessments, and the partial overlap between ALS and depressive symptoms, clinicians often miss the diagnosis of depression, adding to patient distress. This study analyzed data from 180 patients with ALS to develop and validate a nomogram for predicting depression risk using six variables: completion of 9 years of compulsory education, sleep disorders, anxiety, ALSFRS-R total scores, CRP levels, and SIRI values. This tool offers a simple and objective method for identifying patients at a high risk of depression, thereby simplifying the screening process and facilitating clinical decision-making. Depression can arise at any stage of ALS, with the highest risk in the first year post-diagnosis (16.5-fold increase), followed by another elevated-risk period during the second year after motor symptom onset. Previous studies have reported variable prevalence rates of depression in ALS, ranging from 8 to 50%. A major reason for this variability is the difference in measurement tools. A meta-analysis of 46 studies estimated an overall prevalence of 34% (15). Depression questionnaires that primarily assess physical symptoms of depression tend to overestimate patients’ psychological suffering, resulting in higher rates of depression. We chose the HAD scale as it is primarily designed to assess hospitalized patients and avoids assessing physical symptoms associated with depression, making it highly accurate (21). In our cohort, the prevalence of depression was 33.9%, aligning closely with the earlier findings.

### 4.1 Education level and depression

In the present study, lower educational attainment was identified as a significant predictor of depression in patients with ALS. This finding aligns with prior research utilizing large population datasets, including the UK Biobank and FinnGen databases, which demonstrated that higher educational levels are causally associated with a reduced risk of depression and anxiety disorders (22). Additional studies have reported similar associations, indicating that individuals with higher levels of education exhibit a lower likelihood of developing depression (23, 24). Moreover, a meta-analysis demonstrated that every extra year of education was associated with a 3% reduction in depression incidence (25). The protective role of education may be attributable to its contribution to cognitive flexibility, emotional regulation, and cooperation, all of which support more resilient psychological functioning and promote long-term mental well-being (22).

### 4.2 Non-motor and motor symptoms associated with depression in ALS

Previous evidence indicates that patients with ALS gradually experience sleep disorders after the onset of the disease and that these disorders are further correlated with depression (26). Sleep disorders are strongly correlated with increased rates of depression, underscoring their interconnected nature (27). Nearly 90% of depressed patients experience impaired sleep quality (28). Previously considered merely a secondary manifestation of depression, sleep disorders are now regarded as predictive prodromal symptoms (29). Our results indicate that the presence of sleep disorders was a significant predictor of depression risk in patients with ALS. Although the exact mechanisms underlying this association remain unclear, inflammatory responses and circadian rhythm disruption are among the proposed contributors (30). Further, patients with ALS exhibit phosphorylated 43-kDa TAR DNA-binding protein aggregates in hypothalamic regions, which may also disrupt sleep patterns (31, 32) and, thus, increase the risk of depression.

Not only do anxiety and depression often co-occur, their symptoms are also highly correlated (33), and these conditions are also very common in patients with ALS (12, 34, 35). Anxiety is an independent predictor of depression (12) that influences the risk of depression, as people with high anxiety levels are more likely to feel stressed (36). When faced with difficult situations, anxious people tend to view the problem pessimistically, allocating more attention to negative information (37), which may be related to reduced cortisol responsiveness due to progressive hypothalamic–pituitary–adrenal (HPA) axis dysfunction (8). Additionally, similar to the pathogenesis of ALS, neuroinflammation, blood–brain-barrier disruption, peripheral immune cell invasion into the CNS, neurotransmission impairment, HPA axis dysfunction, and microglia activation represent interaction pathways between immune systems and psychopathological mechanisms underpinning psychiatric disorders. Neurodegenerative diseases, including amyotrophic lateral sclerosis, and neuropsychiatric disorders, such as anxiety and depression, are considered “two sides of the same coin” because they share the same signaling pathways, molecules, and mechanisms (38, 39). This may provide a partial explanation for the strong correlation between anxiety and depression in patients with ALS, and the observation that anxiety can act as a predictor of depression.

The motor symptoms associated with ALS significantly influence the occurrence of depression. Lower ALSFRS-R total scores are correlated with an increased risk of depression (34, 40–42). The ALSFRS-R total scores objectively assess the progression and overall severity of ALS by systematically evaluating bulbar function, limb motor function, and respiratory function (43). A decrease in the ALSFRS-R total score does not represent the isolated loss of a single function, but reflects the loss of multiple functional domains (including motor, communication, respiratory, and self-care abilities) and overall functional decline (43, 44). As the ALSFRS-R total score decreases, patients experience overall functional decline. The limitations in physical abilities, from independently eating and dressing to relying on others for assistance, can lead to psychological distress and anxiety (45); simultaneously, the progressive loss of motor autonomy typically results in a gradual decline in the ability to perform daily activities, occupational tasks, and recreational activities, which may contribute to the development of depressive symptoms (41). Swallowing difficulties lead to severe psychosocial consequences, including social embarrassment, isolation, reduced self-esteem due to obvious swallowing difficulties, and anxiety and avoidance behaviors related to eating triggered by persistent fear of choking (46). Breathing difficulties further exacerbate emotional distress, triggering sensations of panic and feelings of helplessness (47). As the condition progresses, respiratory dysfunction often requires symptomatic relief through invasive or non-invasive mechanical ventilation. This is a frequently observed phenomenon associated with increased depression risk (48, 49). These factors collectively form a foundation for susceptibility to the onset of depression.

### 4.3 Inflammatory markers associated with depression

CRP level is recognized as a biomarker of both peripheral and central inflammation. Elevated CRP levels are observed in over 29% of patients with depression (50) and are associated with the severity and symptoms of major depressive disorder (51). Prior studies have also identified associations between increased CRP levels and post-stroke depressive symptoms and prognosis, with CRP serving as a predictive marker for post-stroke depression (52). However, the relationship between CRP levels and depression in patients with ALS remains underexplored. ALS is usually accompanied by a systemic inflammatory response; blood CRP levels are significantly elevated and correlated with disease progression (53). High CRP concentrations increase blood–brain barrier permeability, leading to microglia activation in the central nervous system. The CRP plays a role in regulating and amplifying the inflammatory process and is involved in neuroinflammation (54), which may be a mechanism for depression in patients with ALS. As systemic inflammation in ALS may be a potential therapeutic target, a better understanding of the relationship between peripheral inflammation and depression is clinically important.

In contrast, systemic inflammatory biomarkers, such as SII and SIRI, have been shown to significantly influence the risk of depression (55). The present study demonstrated that SIRI values are a reliable predictor of depression in ALS. The SIRI quantifies the ratio of innate immune effector cells (neutrophils, monocytes, and platelets) to adaptive immune lymphocytes (56–58), reflecting immunological balance. SIRI values incorporate activated circulating immune cells (particularly monocytes included in its calculation) that are known to undergo trans-endothelial migration, thereby promoting microglial activation and subsequent neuroinflammation; SIRI values exemplify the “cross-talk” between central and peripheral inflammation (59). Peripheral blood mononuclear cells are involved in disease progression in patients with ALS (60). Compared to other markers, such as NLR and PLR, SIRI demonstrates superior reliability and representativeness. These strengths underscore SIRI’s potential as a more effective diagnostic marker for identifying depressive symptoms in patients with ALS.

### 4.4 Strengths and clinical implications of the risk model

Depression adversely impacts disease progression and quality of life in patients with ALS. Numerous studies have explored depression-related factors in ALS (9, 34, 42), but this study presents the first validated depression risk prediction model for this population. By integrating both clinical features and routine laboratory indicators, the model provides a practical and comprehensive approach for risk assessment.

The model demonstrated a strong predictive performance, with an AUC of 0.892, indicating excellent discrimination. Calibration analysis showed close agreement between predicted and observed outcomes, as the calibration curve approximated the ideal diagonal. Moreover, DCA and clinical impact curves further substantiated the model’s clinical applicability. The accompanying scoring tables enable rapid identification of at-risk patients in routine practice. These findings support the use of this model for early detection, risk stratification, and timely psychosocial intervention, thereby contributing to improved patient outcomes. This evidence-based tool offers clinicians a practical resource to identify and manage depression risk in patients with ALS.

### 4.5 Limitations and future directions

Some limitations of this study warrant mention. First, the study’s single-center, case–control design and limited sample size may restrict generalizability. Second, due to ethnic and genetic variations in depression, the findings may primarily apply to Chinese populations and may involve selection bias. Third, the retrospective design introduces the possibility of recall bias and inconsistencies due to reliance on existing records. Fourth, among the patients from whom we collected data, three were excluded because they carried gene mutations associated with ALS, seven because they had severe acute inflammatory diseases, three because of hematological abnormalities, and two because they had suicidal tendencies. Patients with ALS carrying gene mutations differ from patients with sporadic ALS in terms of pathophysiological mechanisms, disease progression, and pathological characteristics (61). We chose to exclude patients with gene mutations to improve the homogeneity of the study population and better control for confounding factors. Patients with ALS with comorbid metabolic disorders, neoplastic diseases, severe acute inflammation, or hematological abnormalities exhibit significantly reduced accuracy in laboratory markers (CRP, NLR, MLR, SII, and SIRI), and due to comorbidities, may face greater psychological stress, increasing their risk of depression. We excluded patients with a history of persistent sadness, persistent frustration, severe emotional distress, suicidal tendencies to highlight the ALS-specific depression. Since the above-mentioned groups of patients accounted for a low proportion of our cohort, and to enhance the homogeneity of enrolled patients, improve model accuracy, we excluded the above-mentioned groups. However, excluding such patients introduces selection bias, reducing the model’s applicability to this population. Therefore, future research should further explore the above-mentioned groups. Fifth, we found that the ALSFRS-R total score is a predictor of depression in ALS patients, but we did not analyze the relationship between bulbar subscore, the limb motor subscore, and respiratory subscore, and depression. Considering that the aim of this study was to suggest an ALS depression risk prediction model using simple and easily obtainable clinical and experimental data, we chose the ALSFRS-R total scores, which better represents disease progression and overall functional decline, for analysis and did not perform further subgroup analysis, however, further refinement in this area is required in the future.

## Data Availability

The raw data supporting the conclusions of this article will be made available by the authors, without undue reservation.
